# Genetic analysis of 62 Chinese families with Duchenne muscular dystrophy and strategies of prenatal diagnosis in a single center

**DOI:** 10.1186/s12881-019-0912-x

**Published:** 2019-11-14

**Authors:** Jingjing Zhang, Dingyuan Ma, Gang Liu, Yuguo Wang, An Liu, Li Li, Chunyu Luo, Ping Hu, Zhengfeng Xu

**Affiliations:** Department of Prenatal Diagnosis, Nanjing Maternity and Child Health Care Hospital, Women’s Hospital of Nanjing Medical University, 123# Tianfei Street, Qinhuai District, Nanjing, 210004 China

**Keywords:** Duchenne muscular dystrophy, Dystrophin gene, Multiplex ligation-dependent probe amplification, Next-generation sequencing, Prenatal diagnosis

## Abstract

**Background:**

Duchenne muscular dystrophy (DMD) is a severe X-linked recessive neuromuscular disorder. Patients with DMD usually have severe and fatal symptoms, including progressive irreversible muscle weakness and atrophy complicated with gastrocnemius muscle pseudohypertrophy. DMD is caused by mutations in the dystrophin-encoding *DMD* gene, including large rearrangements and point mutations. This retrospective study was aimed at supplying information on our 4-year clinical experience of DMD genetic and prenatal diagnosis at the Department of Prenatal Diagnosis in Women’s Hospital of Nanjing Medical University.

**Methods:**

Multiplex ligation-dependent probe amplification (MLPA) was used to detect the exon deletions or duplications. And Ion AmpliSeq™ panel for inherited disease was used as the next-generation sequencing (NGS) method to identify the point mutations in exons of *DMD* gene, but the introns were not sequenced.

**Results:**

In this study, the large deletions and duplications of *DMD* gene were detected in 32 (51.6%) of the 62 families, while point mutations were detected in 20 families (32.3%). The remaining 10 families with a negative genetic diagnosis need to be reevaluated for clinical symptoms or be detected by other molecular methods. Notably, six novel mutations were identified, including c.412A > T(p.Lys138*), c.2962delT(p.Ser988Leufs*16), c.6850dupA (p.Ser2284Lysfs*7), c.5139dupA (p.Glu 1714Argfs*5), c.6201_6203delGCCins CCCA(p.Val2069Cysfs*14) and c.10705A > T (p.Lys3569*). In 52 families with positive results, 45 mothers (86.5%) showed positive results during carrier testing and de novo mutations arose in 7 probands. The prenatal diagnosis was offered to 34 fetuses whether the pregnant mother was a carrier or not. As a result, eight male fetuses were affected, three female fetuses were carriers, and the remaining fetuses had no pathogenic mutation.

**Conclusions:**

This study reported that MLPA and NGS could be used for screening the *DMD* gene mutations. Furthermore, the stepwise procedure of prenatal diagnosis of *DMD* gene was shown in our study, which is important for assessing the mutation type of fetuses and providing perinatal care in DMD high-risk families.

## Background

Duchenne muscular dystrophy (DMD) is a severe neuromuscular disease of childhood, defined as progressive deterioration of muscle tissue and resultant weakness, which affects 1 in 3600–6000 male live births [1, 2]. It is an X-linked recessive disease caused by mutations in the *DMD* gene. This gene spans a genomic range of 2.2 Mb and contains 79 exons and 8 promoters. *DMD* gene encodes a protein of dystrophin–glycoprotein complex, which bridges the inner cytoskeleton and the extracellular matrix. The remarkably reduced levels or absence of dystrophin protein is caused by mutations of *DMD* gene, resulting in chronic muscle damage and eventually loss of muscle function in patients with DMD [3].

Many mutations have been described since the discovery of *DMD* gene in 1986 [4]. The mutational studies have indicated that deletions or duplication of exons accounts for approximately 65% of all mutations [5, 6]. Multiplex ligation-dependent probed amplification (MLPA) has been developed to detect the large fragments of deletions or duplications spanning one or more exons [7]. The remaining anomalies are thought to be caused by point mutations, mainly nonsense, frameshift mutations, and small deletion and insertion mutations [8, 9]. The traditional Sanger sequencing of 79 exons of *DMD* gene is costly and time-consuming, making it no appropriate for the purpose of detecting point mutations in *DMD* gene. The target next-generation sequencing technology (NGS), known as deep sequencing, has been widely applied to detect all types of mutations in the *DMD* gene [10, 11].

In the present study, clinical experience of stepwise diagnosis of 62 families with a history of DMD coming to our center in the last 4 years were reported. Firstly, the proband in the families was analyzed using MLPA and NGS. Secondly, the mother carrier status in the families was investigated. Finally, the prenatal diagnosis of fetuses was offered to the pregnant mother.

## Methods

### Patients

**Fig. 1:**
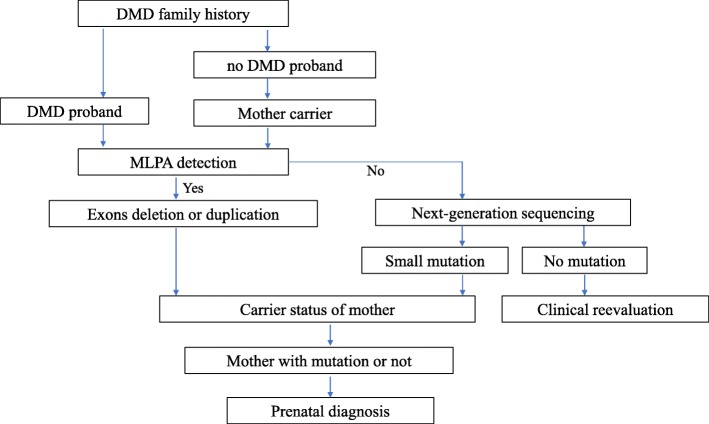
Flowchart for prenatal diagnosis of DMD

The study was performed at the Department of Prenatal Diagnosis in Women’s Hospital of Nanjing Medical University between 2015 and 2018. A total of 155 individuals coming from 62 families with a history of DMD were included in the present study. The female members of the 62 families were in pregnancy or preparing for pregnancy, and 34 pregnant women were referred for the prenatal diagnosis of DMD. The workflow reflected the procedures of the patient management in our study (Fig. 1). This study was approved by the ethics committee of Women’s Hospital of Nanjing Medical University. Written informed consent was obtained from the patients or their guardians.

### Genomic DNA samples

A total of 2 mL of peripheral blood was collected from patients to obtain genomic DNA. For the pregnant women, 10 mL of amniotic fluid was collected at 18–24 weeks gestation for prenatal diagnosis. Genomic DNA was extracted according to the protocol of the QIAamp DNA Mini Kit (Qiagen, Hilden, Germany).

### Multiplex ligation-dependent probe amplification

The SALSA MLPA P034 and P035 kits (MRC-Holland, Amsterdam, Netherlands) were used to detect the deletion or duplication of *DMD* gene. After denaturation, hybridization, ligation, and amplification, the products were examined using an ABI 3500Dx genetic analyzer (Thermo Fisher, USA). The initial data were analyzed using the Coffalyser software V8.0 (http://www.mlpa.com/coffalyser). The 30% increase or decrease in the relative peak area of the probe revealed the duplication or deletion of the exons of *DMD* gene, respectively.

### Next-generation sequencing (NGS)

NGS was used for the point mutation analysis of *DMD* gene in the patients and their family, which was performed using Ion AmpliSeq™ panel of 382 inherited disease-associated genes on an Ion Torrent Personal Genome Machine platform (Life Technologies, MA, USA). Briefly, the Ion AmpliSeq Inherited Disease Panel (Life Technologies, MA, USA) employs over 10,000 primer pairs in just 3 tubes to amplify the exons of 328 genes. After amplification, the library of the target exons was prepared using the Ion Ampliseq Library Kit 2.0 (Life Technologies, MA, USA). The emulsion polymerase chain reaction was carried out using the Ion OneTouch System and Ion PGM Hi-Q View OT2 Kit (Life Technologies, MA, USA)). Then, the template-positive Ion Sphere particles were enriched using the Dynabeads MyOne Streptavidin C1 Beads and washed using the Ion OneTouch Wash Solution (Life Technologies, MA, USA)). Sequencing was performed with the PGM system using the Ion PGM Hi-Q View Sequencing Kit and Ion 318 Chip V2 (Life Technologies, MA, USA) according to the manufacturer’s protocol. Two samples were tested per run. All the sequencing data were analyzed with the standard Ion Torrent Suite software 4.2. The human genome GRCh37/hg19 was used as a reference, and all the detected variants were filtered against dbSNP142. The sequencing results were viewed using the Integrated Genomic Viewer. In addition, Sanger sequencing was used to confirm the most likely disease-causing variants.

## Results

### Exons deletion or duplication of DMD gene was detected by MLPA

Among the 62 families with a history of DMD, exon deletion or duplication was detected in 32 families (32/62, 51.6%) using MLPA (Table 1). The rearrangements contained 31 large deletions and 1 large duplication, accounting for 50% (31/62) and 1.6% (1/62) of families involved in this study, respectively. Single-exon deletions (5/62, 8.1%), which have been verified by RT-PCR, were the most common types of deletions, followed by six-exon deletions (3/62, 4.8%). Exon 49 and 50 were the most frequently deleted exons in 32 families with exon rearrangements.

**Table 1:** The mutation spectrum of DMD patients in 52 families

Family number	Exon ID	Base change	Effect	Mutation type	Carrier status of mother	Status
1	Exon66	c.9568C > T	p.Arg3190*	Nonsense	Yes	Reported
2	Exon47	c.6804_6807delACAA	p.Lys2268Asnfs*2	Small deletions	Yes	Reported
3	Intron65	c.9564-427 T > G		Splicing	No	Reported
4	Exon22	c.2929C > T	p.Gln977*	Nonsense	Yes	Reported
5	Exon7	c.583C > T	p.Arg195*	Nonsense	Yes	Reported
6	Exon41	c.5899C > T	p.Arg1967*	Nonsense	Yes	Reported
7	Exon40	c.5591_5592insT	p.Leu1864phefs*24	Small insertions	Yes	Reported
8	Exon40	c.5697delA	p.Lys1899Asnfs	Small deletions	Yes	Reported
9	Exon16	c.1978_1979delAA	p.K660Efs*59	Small deletions	Yes	Reported
10	Exon14	c.1652G > A	p.W551*	Nonsense	Yes	Reported
11	Exon14	c.1615C > T	p.Arg539*	Nonsense	Yes	Reported
12	Intron45	c.6615-2A > G		Splicing	Yes	Reported
13	Exon6	c.412A > T	p.Lys138*	Nonsense	Yes	Novel
14	Exon56	c.8299G > T	p.E2767*	Nonsense	Yes	Reported
15	Exon62	c.9204_9207delCAAA	p.Asn3068Lysfs	Small deletions	Yes	Reported
16	Exon23	c.2962delT	p.Ser988Leufs*16	Small deletions	Yes	Novel
17	Exon75	c.10705A > T	p.Lys3569*	Nonsense	Yes	Novel
18	Exon47	c.6850dupA	p.Ser2284Lysfs*7	Small insertions	Yes	Novel
19	Exon36	c.5139dupA	p.E1714Rfs*5	Small insertions	Yes	Novel
20	Exon43	c.6201_6203delGCCinsCCCA	p.Val2069Cysfs*14	Small deletions and insertions	Yes	Novel
21	Exon8–17			Dup	Yes	Reported
22	Exon12–29			Del	Yes	Reported
23	Exon48–50			Del	Yes	Reported
24	Exon47–50			Del	No	Reported
25	Exon49–52			Del	Yes	Reported
26	Exon49–50			Del	Yes	Reported
27	Exon46–52			Del	Yes	Reported
28	Exon45–47			Del	Yes	Reported
29	Exon46–48			Del	Yes	Reported
30	Exon1–44			Del	Yes	Reported
31	Exon46–52			Del	Yes	Reported
32	Exon1–60			Del	Yes	Reported
33	Exon47–50			Del	Yes	Reported
34	Exon49–52			Del	Yes	Reported
35	Exon45			Del	Yes	Reported
36	Exon51–54			Del	No	Reported
37	Exon51–54			Del	No	Reported
38	Exon10–11			Del	Yes	Reported
39	Exon18–41			Del	Yes	Reported
40	Exon17			Del	Yes	Reported
41	Exon51–55			Del	No	Reported
42	Exon45–50			Del	Yes	Reported
43	Exon48–50			Del	Yes	Reported
44	Exon50			Del	No	Reported
45	Exon45–50			Del	No	Reported
46	Exon45			Del	No	Reported
47	Exon50			Del	Yes	Reported
48	Exon45–52			Del	No	Reported
49	Exon31–44			Del	Yes	Reported
50	Exon49–51			Del	Yes	Reported
51	Exon43–44			Del	Yes	Reported
52	Exon45–50			Del	Yes	Reported

### Point mutations of DMD gene were identified by NGS

**Fig. 2:**
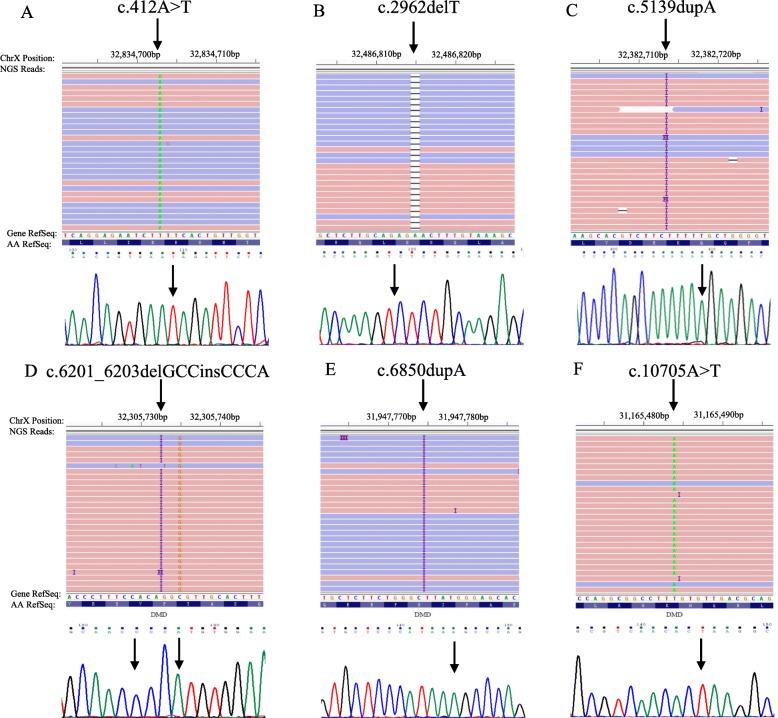
Novel mutations were detected using NGS and verified by Sanger sequecing. NGS reads were shown on the Integrative Genomics Viewer. **a** showed the novel mutation of c.412A > T; **b** showed the novel mutation of c.2962delT; **c** showed the novel mutation of c.5139 dupA; **d** showed the novel mutation of c.6201_6203 delGCCinsCCCA; **e** showed the novel mutation of c.6805dupA; **f** showed the novel mutation of 10705A > T

The remaining 30 families with negative MLPA results were further investigated using NGS. Point mutations were identified in 20 families (20/62, 32.3%), including 9 (9/62, 14.5%) nonsense mutations, 5 (5/62, 8.1%) small deletion mutations, 3 (3/62, 4.8%) small insertion mutations, 2 (2/62, 3.2%) splicing mutations, and 1 (1/62, 1.6%) small deletion and insertion mutation (Table 1). Of all point mutations, six novel mutations were identified in six unrelated families (Fig. 2). All the six novel mutations led to premature termination codons, which was expected to produce truncated dystrophin protein (Table 2). In addition, 10 families had a negative result from findings of MLPA and NGS. Therefore, clinical reevaluation or tests using other molecular methods would be required to achieve a genetic diagnosis for the probands in those 10 families.

**Table 2:** The novel mutations diagnosed by NGS

Familiy number	Age of proband (years old)	CK value (U/L)	ExonID	Base change	Effect	Mutation type	Carrier status of mother
13	8	8616	Exon6	c.412A > T	p.Lys138*	Nonsense	Yes
16	3	12,817	Exon23	c.2962delT	p.Ser988Leufs*16	Small deletions	Yes
17	1	9880	Exon75	c.10705A > T	p.Lys3569*	Nonsense	Yes
18	10	19,964	Exon47	c.6850dupA	p.Ser2284Lysfs*7	Small insertions	Yes
19	11	4880	Exon36	c.5139dupA	p.E1714Rfs*5	Small insertions	Yes
20	5	6179	Exon43	c.6201_6203delGCCinsCCCA	p.Val2069Cysfs*14	Small deletions and insertions	Yes

*CK* Creatine kinase

### Mother carrier status and prenatal diagnosis were investigated by MLPA and sanger sequencing

The mother carrier status was analyzed using MLPA or NGS according to the mutation types of the proband. The results indicated that 45 mothers (45/52, 86.5%) were the carriers, whereas 7 mothers (7/52, 13.5%) did not carry the mutation (Table 1). In the 32 families with exon deletions or duplications, 6 mothers did not have the mutations identified from the proband of the family. Whereas, in the 20 families with point mutations, only 1 mother did not carry the mutation.

**Fig. 3:**
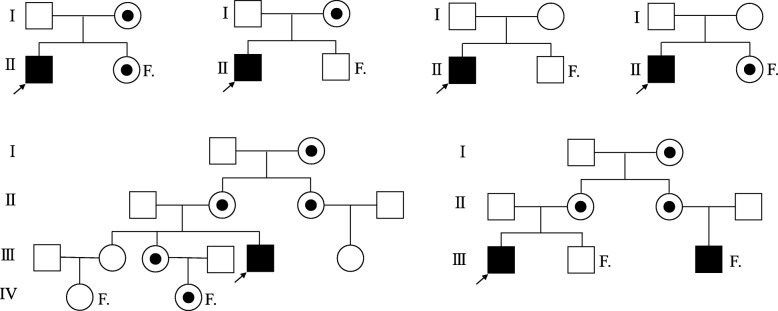
The pedigree patterns of DMD families. ■ and the arrowhead mean the proband of the family. ☉means the carrier in the family. F. means the fetus

According to the results of proband and their mothers, 34 fetuses were tested when the mothers had another pregnancy. Due to the possible germline mosaicism, prenatal diagnosis was suggested to the mothers whether they carried pathogenic mutations or not. The results of prenatal diagnosis showed that eight male fetuses carried the same mutations as the proband, three female fetuses had a carrier status, and the remaining fetuses carried no pathogenic mutation (Table 3). According to the family information and the mutation results, we summarized the pedigree patterns of 62 DMD families (Fig. 3).

**Table 3:** Prenatal diagnosis of 34 fetuses in high-risk families with DMD family history

Family number	Proband	Carrier status of mother	Fetus	Pregnancy outcome
1	c.9568C > T	Yes	–	–
2	c.6804_6807delACAA	Yes	–	–
2	c.6804_6807delACAA	Yes	+	Termination
3	c.9564-427 T > G	No	–	–
10	c.1652G > A	Yes	+	Termination
12	c.6615-2A > G	Yes	–	–
13	c.412A > T	Yes	–	–
14	c.8299G > T	Yes	–	–
15	c.9204_9207delCAAA	Yes	+	Termination
21	Exon8–17	Yes	–	–
23	Exon48–50	Yes	–	–
24	Exon47–50	No	–	–
25	Exon49–52	Yes	–	–
26	Exon49–50	Yes	+	Termination
27	Exon46–52	Yes	+	Termination
28	Exon45–47	Yes	+	Termination
31	Exon46–52	Yes	Carrier	–
33	Exon47–50	Yes	–	–
34	Exon49–52	Yes	–	–
36	Exon51–54	No	–	–
37	Exon51–54	No	–	–
39	Exon18–41	Yes	–	–
40	Exon17	Yes	–	–
41	Exon51–55	No	–	–
42	Exon45–50	Yes	–	–
44	Exon50	No	–	–
45	Exon45–50	No	–	–
46	Exon45	No	–	–
47	Exon50	Yes	Carrier	–
48	Exon45–52	No	–	–
49	Exon31–44	Yes	+	Termination
50	Exon49–51	Yes	+	Termination
51	Exon43–44	Yes	Carrier	–
52	Exon45–50	Yes	–	–

## Discussion

Many patients with a family history of genetic diseases have visited our center. If a proband was confirmed in the family, the members of the family would be worried about their next generation and strongly wished to determine the genetic causes to avoid having the fetus with the same disease. In the present study, data from patients with DMD were retrieved and analyzed. A total of 155 individuals deriving from 62 families with a history of DMD were included in the study. In all, 32 different large rearrangements and 20 point mutations including 6 novel mutations, were identified in 62 probands of the families using MLPA and NGS.

MLPA has been used to detect the large rearrangements of *DMD* gene since its discovery by Schouten in 2002 [12]. To date, it was reported that approximately 65% patients showed exon deletions or duplications in the *DMD* gene [5, 6]. The results of the present study demonstrated that 51.6% of the probands had large rearrangements, which was slightly lower than that in the similar studies (70.5% [7], 71% [13], 78.7% [14]). The remaining mutations were detected using NGS, and point mutations accounted for 32.3% of the molecular pathology in *DMD* gene in our study, which was slightly higher than that in other studies (30%) [8] (Table 1). It was believed that these differences could be due to the sample size and sample source. In China, MLPA has been widely used in many hospitals and laboratories, while NGS is applied in only a few centers because of its high requirement for laboratory conditions and complex data analysis. In our center, the *DMD* gene could be tested using not only MLPA but also NGS. Therefore, many patients visited the center for detecting point mutations when their MLPA results were negative in other laboratories. As a result, the present study showed a lower rate of rearrangement and a higher rate of point mutations, compared with the findings of the other studies of DMD.

Further, many novel point mutations could be found in the *DMD* gene using NGS. Wang et al. reported that about one third of the mutations could be firstly identified in patients with DMD [8]. This study reported six novel mutations in patients with DMD (Table 2). All the six novel mutations (c.412A > T, c.2962delT, c.10705A > T, c.6850dupA, c.5139 dupA and c.6201_6203delGCCinsCCCA) led to a premature stop codon, resulting in the production of a shortened dystrophin protein, which could harm the normal function of dystrophin.

To date, no effective therapies are available for patients with DMD. Therefore, families with a history of DMD showed anxiety and strongly requested prenatal diagnosis to avoid having another child with DMD. The proband’s mother and other related female family members were suggested to accept genetic counseling and carrier testing. In the present cohort, the results demonstrated that 45 (45/52, 86.5%) mothers were carriers. If the mother was not a carrier, her son with DMD mostly had a de novo mutation. However, germline mosaicism in DMD should not be ignored as described as early as in the 1980s [15, 16]. Wang et al. reported 3 cases of germline mosaicism in 50 mothers with no pathogenic mutations, suggesting that mosaicism should be considered in the genetic counseling. In our center, prenatal diagnosis was recommended to pregnant women whether they carried the disease-causing mutations or not. In the present study, a total of 34 pregnant women accepted the prenatal diagnosis. The results demonstrated that eight male fetuses were affected, three female fetuses were carriers, and the remaining were normal for the identified mutations in the families respectively (Table 3).

In this study, 10 families did not have positive mutations according to the complete analysis of the exons of the *DMD* gene. Besides *DMD* gene, the exons of 381 inherited disease-associated genes in the disease panel were negative for pathogenic mutations in our study. These genes are associated with over 700 inherited diseases including some other neuromuscular diseases. The detailed diseases are shown in the web: lifetechnologies.com/ ampliseqready. Therefore, the clinical diagnosis should be firstly revised in further research. If the manifestations and muscle biopsies were consistent with DMD, the introns of the *DMD* gene should be comprehensively investigated. If the results for introns of DMD gene were still negative, the other muscle disease panels or whole-genome sequencing could be used to analyze the pathogenic mutations.

## Conclusions

DMD causes great harm to many patients and their families due to the lack of effective therapies. Accurate genetic and prenatal diagnoses are urgently needed for the families with a history of DMD to avoid having children with DMD. In this retrospective study, MLPA and NGS were combined for the genetic analysis and prenatal diagnosis of 62 Chinese families with DMD proband. Meanwhile, six novel pathogenic point mutations in the *DMD* gene were reported. Generally, when the family members with a history of DMD came to the center, MLPA was first performed for detecting exon deletions or duplications. If the MLPA results were negative, NGS was used to detect the mutations. If positive results were obtained, the carrier testing of the mother was performed. Considering germline mosaicism, the prenatal diagnosis was proposed to all the pregnant women in these families whether they carried the disease-causing mutations or not. If the mutation can not be determined using MLPA and NGS, the clinical information should be evaluated and other methods could be used to uncover the underlying pathogenic mutations (Fig. 1).

## Data Availability

The datasets generated and/or analysed during the current study are not publicly available because the raw MLPA and sequencing data were too large to upload but available from the corresponding author on reasonable request.

## Abbreviations

CK: Creatine kinase
DMD: Duchenne muscular dystrophy
MLPA: Multiplex ligation-dependent probe amplification
NGS: Next-generation sequencing
